# Accelerating innovation in sustainable development through transdisciplinary impact research

**DOI:** 10.1016/j.isci.2026.115448

**Published:** 2026-03-23

**Authors:** Rebekah Brown, Brett Davis, Matthew French, Tony Wong, Diego Ramirez-Lovering, Steven L. Chown, Thomas Clasen, David Johnston, Ansariadi Ansariadi, Amelia Turagabeci, Kerrie Burge, Chris Greening, David McCarthy, Stephen Luby, Karin Leder, the RISE Consortium

**Affiliations:** 1 Australian National University, Canberra, ACT 2600, Australia; 2 Monash Sustainable Development Institute, Monash University, Melbourne, VIC 3800, Australia; 3 Office of the Deputy Vice-Chancellor (Research), Monash University, Melbourne, VIC 3800, Australia; 4 Monash Art Design and Architecture, Monash University, Melbourne, VIC 3145, Australia; 5 School of Biological Sciences, Monash University, Melbourne, VIC 3800, Australia; 6 Rollins School of Public Health, Emory University, Atlanta, GA 30322, USA; 7 Centre for Health Economics, Monash University, Melbourne, VIC 3145, Australia; 8 Faculty of Public Health, Hasanuddin University, Makassar, Indonesia; 9 School of Public Health and Primary Care, Fiji National University, Tamavua, Suva, Fiji; 10 Biomedicine Discovery Institute, Monash University, Melbourne, VIC 3800, Australia; 11 Department of Civil Engineering, Monash University, Melbourne, VIC 3800, Australia; 12 Woods Institute for the Environment, Stanford University, Stanford, CA 94305, USA; 13 School of Public Health and Preventive Medicine, Monash University, Melbourne, VIC 3004, Australia

**Keywords:** global change, interdisciplinary application studies

## Abstract

Many global sustainable development challenges remain intractable due to the inadequacy of traditional approaches for dealing with complex, multi-dimensional, systemic problems from which the challenges arise, and the absence of effective innovation pathways between discovery and development practice. Faster paced innovation and systems change to overcome poor progress on the sustainable development goals (SDGs), particularly for the global South, where a lack of cost-effective and accessible solutions results in political paralysis. In this context, mission-based, transdisciplinary impact research approaches that fast-track methodological development of research and practice in sustainable development are highly promising for achieving systems change and evidence-informed solutions to global sustainability challenges. Here, based on learnings from a large-scale program applying such an approach and comparable global exemplars, we provide some essential “pillars” of support as foundational practical guidance for others to implement this enabling model for impact-oriented research while concurrently delivering real-world impact and legacy.

## Graphical abstract

**Figure:**
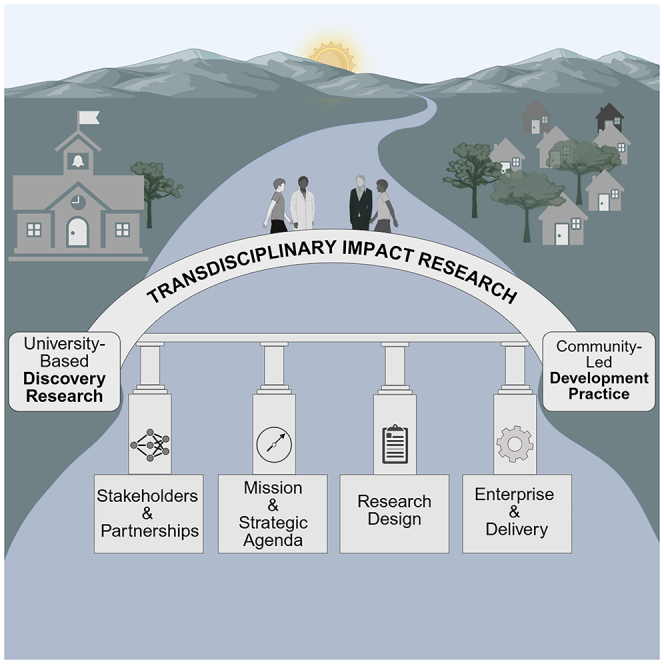

## Introduction

Many of the most pressing sustainable development challenges are global and complex, encompassing virtually all dimensions of people and planet, from energy and environment to poverty, health and economy.^1^ This high dimensionality, and the complex interdependence and inseparability of the ecological, environmental, economic, and social systems from which the challenges have arisen,^2^ has made it increasingly difficult to make effective progress on key measures such as the sustainable development goals (SDGs).^3^

In many regions of the global South, key indicators of health and wellbeing under the SDGs have stagnated or reversed over recent years,^4^ despite the period seeing record investment in development aid.^5^ This situation points to the inability of traditional development approaches to effectively address or even keep pace with development needs, and the intractability of many sustainable development challenges. There is also emerging evidence that many accepted practices may not be achieving the desired outcomes because they do not effectively address the complex multi-dimensional nature of the problems that underpin these persistent development challenges.^6^^,^^7^ From climate, water, sanitation, food, environment and ecology, to poverty, education, employment, trade and politics; the complex interdependence of the social, ecological, environmental, economic and political systems underpinning the challenges^2^ also make it difficult to formulate and test effective solutions. While misalignment of political will and priorities are often cited as major reasons for the slow pace of progress toward the SDGs,^8^ it is also clear that prevailing practices and systems are inadequate to meet these complex, systemic challenges or catalyze effective innovation.

The need for a different approach to address the SDG challenge was the subject of the September 2021 report of the international science council (ISC), *unleashing science: delivering missions for sustainability*,^9^ which advocated for a new research paradigm focused on ambitious, long-term, goal-focused science missions. This is mirrored in the broader research-funding landscape, which has seen a move toward long-term missions such as the Horizon Europe^10^ Missions program and Japan’s Moonshot^11^ program. The ISC’s paradigm shift aims to bring discovery research and development practice closer together for deeper and faster innovation to expedite progress on the SDGs over the coming decade.

This requires these problems to be conceptualized in ways that capture and address this multi-dimensionality and complexity, acknowledging the interdependence and inseparability of human and environmental health and the systems in which they exist, to develop holistic sociotechnical “planetary health”^12^ responses. Universities have a potent but largely unrealized role to play in supporting and driving innovation in the development sector, by bringing to these major development challenges new ideas, discovery approaches, and collaborations across diverse disciplines,^13^ and by convening disparate actors in a transdisciplinary space to match the complexity of intractable socio-ecological challenges. It remains an open question, however, whether in practice the right conditions can be created by academic institutions to enable such transdisciplinary initiatives to produce actionable, scalable results.^14^ With a recognized lack of effective innovation in the development sector and an over reliance on accepted practice^15^ despite evidence of inadequate or declining efficacy,^9^^,^^10^ it is now more urgent than ever to bring the potential of academia to bear on these grand sustainability challenges. What has been missing, we argue, is an enabling mission-oriented framework that bridges the distinct epistemologies of discovery research and development practice in a way that fosters contemporaneous research-to-practice translation and so provide field-proven and actor-accepted innovations to fill the conspicuous gaps in the sector’s innovation funnel.^16^

The difficulty in achieving the right conditions for such initiatives is understandable given the significant institutionalized barriers that exist across the sectors involved (universities, aid delivery organizations, governments, multilateral organizations), each of whom have different objectives, remits and funding sources and rules. These obstacles alone are substantial, global, and systemic; but the urgency and scale of the need to improve efficacy in development practice place an obligation on the research and development aid communities to collaborate on creating a more efficient innovation pathway from discovery to impact.

## Mission-based transdisciplinary impact research as an enabling framework

Based on our broad experience over more than three decades in designing and delivering research and development programs, we argue that a promising approach for fast-tracking the traditionally long, linear, inefficient, and multi-stage research, translation, and scale-up process is to deliver these activities in parallel, by combining an impact-oriented experimental intervention with a rigorous research framework, supported by core participation of the end users, authorities, and practitioners who will ultimately benefit from and/or scale up the innovation.

However, there are considerable challenges associated with conceptualizing, developing, and operationalizing an impact-oriented experimental intervention at scale within a rigorous research framework, with fundamental and intrinsic tensions between discovery research and intervention implementation in the real world. To resolve these tensions, and expand the ambition of such an activity beyond the boundaries of traditional (discovery) research, we look to precedents in transdisciplinary research,^17^^,^^18^ action-research,^19^ implementation research,^20^ complex systems,^21^ and post-normal science.^22^ Each of these concepts speak partially to this challenge. Transdisciplinary research recognizes the vital importance of non-academic actors (beneficiaries, government, industry, and civil society) in designing and implementing research; action-research emphasizes the importance of real-world experimentation involving practitioners in developing scalable solutions; implementation research focuses on how development activity is best conducted; complex systems research attempts to recognize and capture the entire complexity of the challenge; and post-normal science acknowledges the irreducible uncertainty in complex sustainability transformations and the need for diverse knowledge and values beyond academic expertize. The concept of transformative transdisciplinary research^23^ recognizes the importance of these research lenses and in particular the personal and relational transformation that is required to deliver programs capable of transformative change.

Here, we specifically consider the challenge of bringing academic expertize and research funds mobilization to bear on complex and intractable challenges in sustainable development practice, and in our case how these precedents have applied to the revitalizing informal settlements and their environments (RISE) program (https://www.rise-program.org/).^24^

The RISE program is a cluster randomized controlled trial (RCT) of a neighborhood-scale, community-centred, sociotechnological approach to achieving enduring improvement to living conditions and therefore community health in urban informal settlements by co-designing and implementing “acupunctural” neighborhood-scale sanitation and drainage improvements based on water-sensitive and planetary health principles. The trial involved 24 participating communities in Fiji and Indonesia comprising more than 9,000 residents and 1,700 households. The RCT was designed to systematically and empirically research the linkages between human health, wellbeing, and environmental systems in informal settlements, and involves the full implementation of neighborhood-scale upgrades to a randomized half of the 24 settlements as part of the RCT. The RISE program therefore serves as a useful experiential example of an international research program resourced to explicitly solve a global sustainability challenge at scale involving significant political, regulatory and community engagement and support. A more detailed description of the RISE program and its outcomes are provided in supplemental information.

Our interest with RISE was how to create an effective pathway between an idea originating in academia and a scalable solution that is ready to upscale within the time frame of a traditional academic project. This resulted in the conceptualization of transdisciplinary “impact” research (for convenience, TIR), which integrates a mission-oriented (or challenge-based) programmatic activity, such as implementation of an experimental sociotechnical intervention at scale, within a robust research framework, to effectively and simultaneously meet the expectations and requirements of both academic and development (impact) funding.

The explicit design intent of such a mission is to conduct “*in situ*” science and implementation in real-world environments at the intervention’s intrinsic scale, capturing a multi-faceted evidence base and training local end-users, authorities, practitioners and other actors who will ultimately drive local translation through active participation, and at the same time embedding capacity building, dissemination and policy change within the research program’s core delivery to enable scale-up once evidence of the efficacy of the intervention is delivered.

Delivering the core intervention at the required scale—in the case of RISE, 24 informal neighborhoods covering approximately 1,700 households in two countries—requires access to significant additional funding and resources beyond the traditional research regime funded by academic grants, crossing into a donor-controlled development-aid space typified by high stakes (lives and livelihoods), high uncertainty (political, technical, and financial), diverse values (research, donor, government, and community actors), and short time frames and high urgency (interdependent research and implementation time frames). TIR is therefore highly transdisciplinary^25^^,^^26^^,^^27^ by its nature, positioned not just between, across, and beyond individual academic disciplines and the integration of multiple sectors and stakeholders as active participants, but also through its interaction with and modification of the real world at scale to simultaneously create systems change, realize a meaningful development impact (and so satisfy the development donor), and train a cohort of practitioners and authorities to support independent, self-sustaining scale-up. The approach is consistent with and complements the concept of transformative transdisciplinary research, emphasizing the core imperative for the project to be co-designed with participants and beneficiaries and local leadership; here, we offer insights into how a large-scale impact activity can be integrated by academic institutions as the central experimentation and evidence engine.

TIR essentially serves as a bridge between the archetypal extremes of “discovery research” and “development practice”. Traditional discovery research focuses on testing ideas and theories in highly controlled systems and/or using highly rationalized datasets to meet a predetermined level of scientific validity,^28^^,^^29^ and is funded explicitly for research and rarely for implementation at scale.^30^ Development practice, on the other hand, addresses identified problems or needs by applying best practice based on well-established solutions, and draws on a development funding pool that focuses on delivered impact where interventions are generally assessed using a results framework for outcome and impact without scientific rigor. A TIR initiative therefore needs to be at once a robust, fundable research program and a fundable, impact-driven development program, while balancing and reconciling the institutional norms, requirements, expectations, evaluation frameworks, and timelines of both. Since TIR programs must encompass research, action (implementation), and capacity building in equal measures, they will often be positioned outside the typically strict funding mandates of both research and development grant sources.^31^ The funding, design, and operational complexities that both researchers and practitioners face must be recognized, embraced and overcome.

Drawing on the authors’ broad experience of designing, operationalizing and implementing the RISE program, we have identified four key *pillars* of support that are needed to create the conditions for designing and executing TIR as a translational bridge between discovery research and development practice. Table 1 summarizes these pillars of TIR and compares them with the archetypal end-members of discovery research and development practice. Examples are then discussed.

**Table 1:** Reconciling discovery research with development practice: key pillars underpinning transdisciplinary impact research

Pillars		Discovery ⇨	Transdisciplinary impact research	⇦ Development practice
Pillar 1: Stakeholders and partnerships	stakeholders	limited, selected, controlled; often peers have similar alignment and are partnered through institutional collaboration arrangements	co-design; high stakes; critical roles; open, uncontrolled, participatory, diverse and sometimes conflicted alignment with unclear interests	limited, well-defined, context specific, largely controlled and replicable, with similar alignment, partnered through institutionalized arrangements
	engagement and power relations	short-term; for academic benefit; institutionalized power relations	“win–win” engagement; capacity building, long-term research for impact; strategic local empowerment and explicit normalization of power relations	beneficiary-driven and institutionalized power relations
Pillar 2: Mission and strategic agenda	scope	project-based research to advance academic knowledge	mission-based testing and translation of ideas/solutions with knowledge transfer for future scaling and implementation	project-based delivery of interventions and resources for development impact
	mandate	research only, governed by strict rules	bespoke hybrid of research and implementation, with no direct precedent	implementation only, governed by strict rules
Pillar 3: Research design	dimensionality and complexity	low/narrow, well-defined, closed system	high, semi-controlled, semi-open system	high, uncontrolled, open system
	validity	high, internal; defined study parameters; basis of delivery (outcome)	internal and external, negotiated to balance academic rigor and practicability in a semi-open system	low, external, not required for delivery (outcome)
	unit of analysis (scale)	narrow, targeted, scaled for scientific control	scaled to balance scientific validity *and* delivery objectives	wide and scaled to meet broadest delivery objectives
Pillar 4: Enterprise and delivery	enterprise scale	small and within institutional structures	fit-for-purpose; new or hybrid entity/vehicle with flexible operational structure	small to large; delivered by development actors and contractors
	expertize and enterprise culture	academic, bench-based research and discovery culture	academic and operational capacity-building culture	operational and pragmatic delivery culture

### Pillar 1: Stakeholders and partnerships

Operating in the transdisciplinary “middle” requires enrollment of a range of stakeholders that is broader, deeper, and more diverse than for discovery research or development practice alone—harnessing not just participants but advocates across academia, government, industry, philanthropy, non-profits, and community organizations, with their array of skills, vested interests, expectations, motivations, paces of operation, and priorities.

In a TIR program, stakeholders are not selected but collected as they are encountered during the mission, program design, and implementation, to form an expanding “coalition of support” with whom all aspects of the program must be co-designed. All stakeholders on the critical path need to be recruited to action on behalf of the program through the co-design of their participation and program parameters. Stakeholding requires extraordinary vigilance and attendance, involving (1) pro-active identification of all stakeholders needed for program delivery, (2) continuous soft advocacy to secure political support and stakeholder contributions, (3) co-design and alignment of contributions in time and space, (4) facilitation of consensus building and working relationships across the program, (5) a focus on keeping all stakeholders engaged, empowered, and fulfilling their pledges, and (6) management of the continuous evolution of stakeholder interests and availability.

Achieving the multi-dimensional systems-level change targeted by TIR necessitates that end-users and implementing stakeholders become active participants in the program, to co-design resolutions to obstacles to implementation and to develop the local capacity and ownership that can enable and catalyze future upscaling. This, in effect, short-circuits the traditional research-then-translate model by delivering both research and translational capacity-building simultaneously.

Stakeholder engagement and participation is not just best practice but is the critical enabler and purpose of TIR.^32^^,^^33^ The risks of TIR program failure are numerous, and are mitigated only through genuine and continual stakeholder engagement. The outcome is only made possible by design and solving challenges together, and by securing support and action from key stakeholders as dynamic participants in the real-world experiment.

Tackling these issues in addressing development challenges in low- and middle-income countries (LMICs) also raises specific ethical and moral complexities founded in significant and historical power imbalances^34^^,^^35^ and the inequity of “extractive” research practices.^36^^,^^37^ By enabling active participation by local actors and capacity building for immediate implementation, TIR creates the opportunity to directly address the stark and intrinsic power and capacity imbalance between the global North and South.^38^^,^^39^ The approach also rests on the ability to move beyond the principle of “do no harm”, to add lasting tangible value to the lives of study participants and communities as a direct result of the research endeavor, during its active phase and beyond. Careful balancing is essential to avoid “buying” the research participation of communities through incentives while ensuring sufficient community motivation to remain engaged in co-designed action-research.

As an example of cross-country TIR, the RISE program demonstrates that it is possible and necessary to establish and maintain a fit-for-purpose engagement modality that reconciles the power imbalances across diverse countries and institutions to deliver the TIR program, in a just and ethical way for the benefit of all partners. Drawing on learnings from development practice, the RISE program has financed and established large local teams, offices, labs, and other facilities in collaboration with local universities, and has engaged and trained a multidisciplinary cohort of research, engineering, field, construction, and support personnel to co-design and deliver the program in Fiji and Indonesia. The aim is to have self-perpetuating impact long after the program has ended, by founding long-term partnerships and mission-based learning alliances under a “training of trainers” model. In the participating communities, which ultimately stand to gain (or lose) most in such an endeavor, the RISE program has actively fostered strong enabling relationships and uplifted local capacity and ownership through the establishment of community engagement committees that build on existing community and local governance structures.

The International Center for Diarrheal Disease Research, Bangladesh (https://www.icddrb.org/) is a prime example of a long-running donor-funded “impact research” program to address major public health challenges in Bangladesh—explicitly designed to address capacity and power imbalances by building local skills in health research and practice. The success and longevity of the Center attest to the importance of nurturing and developing context-appropriate expertize and solutions *in situ*. This has been demonstrated to decouple traditional dependency relationships as an effective long-term approach to addressing complex and intractable development challenges.

### Pillar 2: Mission and strategic agenda

In contrast with the traditional project-based orientation of both research and development practice, TIR is guided by a mission to enact change at scale,^40^ coupled with rigorous learning and evaluation, ultimately to find an institutionalized home for global diffusion of proven interventions. The conceptual framework for such mission-based programmatic research has a very different goal from either traditional research or development praxis: it includes the non-negotiable imperative that the participating communities, local partners, and broader stakeholder community are engaged and trained as equals, and that knowledge is transferred in both directions in a way that supports local optimization and independent scaling of successful innovations, along with the upskilling of participants as future practitioners and agents for change.^41^^,^^42^ In the example of the RISE program, the core research funding for the RCT was leveraged to secure funding from development sources for the infrastructure intervention on which the research and capacity building both depend. This required new arrangements and agreements, negotiations with and among funders around complementary commitments, and navigation of the complex interrelationships and competing expectations of the funding stakeholders to meet shared goals.

At a programmatic level, the challenge is to conceive and maintain a program that aligns research-, translation-, and practice-based outcomes, while also ensuring sufficient flexibility for independent research ideation, translational and practice-based learnings to meet the needs and career development trajectories of both researchers and delivery staff. This involves explicit, ongoing navigation of diverse vested interests, working norms, and expectations among participants, donors, and stakeholders, and relies heavily on nurturing a “shared mission” to which all can subscribe. It also requires a cultural shift away from typical short-term project time frames that provide research outputs within a few years to the much longer commitment associated with delivering the “impact” aspect of the program.^24^ These challenges are non-trivial, and there are few precedents among initiatives related to complex development challenges.

### Pillar 3: Research design

Solving complex real-world problems involves dealing with many causal dimensions, dependent and independent variables, stakeholders, cultures, geographies, and actors. Such challenges call for integrated multi-dimensional sociotechnical responses at sufficient scale to achieve system-level change—interventions that must be tested, translated, adapted, contextualized, and proven at the intrinsic scale of the solution. However, the scale and complexity of systems-based interventions typically confound objective assessment and raise the intimidating challenge of how to obtain scientifically valid research outcomes in large real-world interventions.^43^

Bounding this reality typically involves reducing the scope of the intervention to a narrow set of variables that address a small, defined part of the problem to match a highly controlled research architecture; or it can involve application of a narrow disciplinary lens to an inherently complex intervention. To generate the multi-dimensional evidence of the efficacy, practicability, and scalability needed to support downstream uptake and diffusion, however, the research scope needs to match the dimensionality of the problem and the anticipated intervention/solution scale. In the case of the RISE program, the research framework was determined by aligning the conceptual scale of the problem with the intervention, resulting in a multi-community, multi-country, multi-disciplinary program sufficient to support a robust and statistically powered research outcome.^44^ This integration between intervention and research took two years for program conception and development. Exceptionally careful negotiation on the structural terms of the intervention and the research–action relationship was needed to achieve the dual objectives of enacting a real-world development project while enabling application of a scientifically valid assessment.^45^

Coordination between intervention and research is critical both during design and delivery. Hermetic separation between the scientific evaluation and the design and implementation of the intervention does not guarantee validity; without concurrent design of the research and intervention within a unified architecture, the intervention would only by chance have the requisite scale and design to allow independent, scientifically valid assessment. A more robust route to optimizing scientific validity is to co-design the research-intervention framework, and form an agreement on whether there are data that cannot be shared without comprising scientific integrity. For the RISE program, this resulted in co-designed criteria for site selection, site number and spatial size, establishment of the parameters and allowed actions that form the intervention, and an agreed framework for the structure and delivery of the RCT. It has also required active, adaptive and pragmatic management to maintain effective separation of the on-the-ground research and intervention teams for certain aspects where failing to do so would compromise research rigor. For example, research data cannot be shared if it would explicitly alter the intervention design.

The ambitious millennium village project (MVP), implemented across ten villages in Africa between 2005 and 2015, is a notable example of a development intervention that sought to overcome the limitations of piecemeal approaches in an effort to achieve transformative development impact and poverty reduction in the context of the UN’s millennium development goals.^46^ The MVP was a village-scale intervention with high dimensionality (health, education, agriculture, and governance). Unfortunately, reviews have indicated that the opportunity to leverage the initial investment has been limited. This was due, in part, to the lack of an explicit integrated and scientifically rigorous research design implemented in parallel with the intervention. The paucity of rigorously acquired evidence may have limited the broader dissemination and upscaling of the project’s learnings on how to disrupt trajectories of poverty.

### Pillar 4: Enterprise, expertize, and culture

A key pillar of the evidence base supporting effective translation of innovation into development practice is how practicable and cost-effective the new approach is at scale. The intrinsic scale of the problem and the scale needed to produce scientific and statistically powered results must be matched. For any given intervention there is therefore a threshold or minimum scale below which the acquired evidence is unlikely to support downstream uptake and diffusion. For neighborhood-scale interventions this results in an action-research program of significant size (often across multiple communities) and complexity that necessitate new and more flexible organizational structures, financing arrangements, and ways of working, as well as a time horizon of at least five to ten years. Establishing a program of this size needs an enterprise-scale approach that puts sufficient emphasis on operations, in conjunction with research and implementation. For example, the RISE program includes (1) program management, international scientific and development advisory panels, an executive, communications, data management, risk management, and donor relations; and (2) engineers, architects, community engagement workers, builders, accountants, and procurement experts, including large in-country teams, all beyond the core research group.

The World Mosquito Program (WMP; https://www.worldmosquitoprogram.org/) is an exemplar of an enterprise approach to mission-based research and implementation, having taken an idea for dengue control from initial discovery research trials through translation to deployment at scale. As another pioneering program in this bridging space, the WMP started in one small location in northern Australia and is now a fully developed enterprise encompassing governance, management, data management, in-country operations, research, and engagement, with operations across 14 countries covering more than 11 million people. Such impact and scale-up would not have been possible without enterprise-scale operational arrangements that can be an effective bridge between research and development practice enterprise norms, standards, and best practices.

## Conclusion

The fundamental architectures of discovery research and development practice are not easily reconciled, yet bringing these epistemologically and operationally divergent worlds together is essential for effective innovation to achieve the SDGs. TIR in this context provides an enabling framework to support the design, implementation, and funding of transformative programs that deliver both lasting development impact and novel research outcomes to address intractable and complex sustainable development challenges.

As a model that facilitates a sophisticated level of engagement to address complex systems problems, TIR is applicable to a range of settings and theories of change (e.g., energy, food, and climate) where successful translation into practice could be accelerated by innovating at scale. TIR is a potentially powerful enabling model: if recognized and supported more broadly, it could unlock funding streams and opportunities for innovation and rapid translation to scale—not just in the development aid space but across a broad spectrum of targets in sustainable development and beyond.

Navigating, reconciling, and co-creating endeavors in the distinctive bridging space between research and practice in a development context is challenging but rewarding. By codifying our perspective in the four pillars of TIR, we seek to stimulate discussion, debate, testing, and further development by others to advance ambitious mission-based programs for impact. Critically, we argue that it is the research sphere that can take the initiative by expanding the scope of the university enterprise, which stands to gain much by increasing its social return and relevance through leadership in solving systemic development challenges.

## Acknowledgments

The RISE program that forms the basis of this work is funded by the 10.13039/100010269Wellcome Trust, the 10.13039/501100008631New Zealand Ministry of Foreign Affairs and Trade, the 10.13039/501100000996Australian Department of Foreign Affairs and Trade, the Government of Fiji, the 10.13039/100004425Asian Development Bank and 10.13039/501100001779Monash University. The program involves partnerships and in-kind contributions from the City of Makassar, the 10.13039/501100003327Cooperative Research Centres for Water Sensitive Cities, 10.13039/100017522Fiji National University, 10.13039/501100010740Hasanuddin University, Southeast Water, Live and Learn Environmental Education and Melbourne Water.

The Graphical Abstract has been created with permission in BioRender. Lynch, F. (2026) https://BioRender.com/mmcysk2.

## Author contributions

Conceptualization: R.B., B.D., M.F., K.L., S.L., D.R.-L., and T.W.; methodology: S.L.C., D.J., A.A., A.T., K.B., C.G., and D.M.; writing – original draft: B.D., R.B., M.F., and K.L.; writing – review and editing: All authors.

## Declaration of interests

The authors declare no competing interests.
