# Supplementary material for: Accelerating innovation in sustainable development through transdisciplinary impact research
Source: iScience. 2026 Mar 23;29(4):115448. doi: 10.1016/j.isci.2026.115448 (PMC13087720; doi:10.1016/j.isci.2026.115448)
Supplement: Document S1. Supplemental information [file mmc1.pdf]

iScience, Volume *29*

Supplemental information

Accelerating innovation in sustainable development through transdisciplinary impact research

Rebekah Brown, Brett Davis, Matthew French, Tony Wong, Diego Ramirez-Lovering, Steven L. Chown, Thomas Clasen, David Johnston, Ansariadi Ansariadi, Amelia Turagabeci, Kerrie Burge, Chris Greening, David McCarthy, Stephen Luby, Karin Leder, and the RISE Consortium

Supplemental Information

The Revitalising Informal Settlements and their Environments (RISE) program is a cluster randomized control trial (RCT) of a water-sensitive, nature-based approach to improving living conditions in urban informal settlements through the co-design and construction of neighbourhood-scale sanitation, drainage and access upgrades. The RCT involves 24 informal settlements, 12 in Suva, Fiji and 12 in Makassar, Indonesia, which were randomized into balanced intervention and control groups, and covering a total of 1,649 households and 8,153 residents (as at July 2025).

The RISE study hypothesises, from a planetary health perspective, is that the intervention will improve environmental biodiversity and quality, reduce human faecal contamination in the environment, and interrupt faecal–oral transmission leading to reduced intestinal carriage of pathogens and drug-resistant gene markers, concurrently enhancing psychological, social, and economic outcomes and resulting in benefits to human health and wellbeing.

Settlements were selected from a shortlist of over a hundred potential sites on the following criteria: (a) residents representative of the most vulnerable populations; (b) poor water and sanitation services; (c) poor drainage and/or flooding conditions; (d) high risk of water-borne and -related diseases; (e) size (consisting of approximately 30-100 houses); (f) number of children under 5 years of age; (g) land tenure security; (h) no other ongoing or planned interventions; and (i) some physical separation from other settlements with clear physical boundaries (not directly contiguous with neighbouring areas).

The RISE approach is based on the premise that the health of people and the environment are intrinsically linked, interdependent and inseparable, and that human health in informal settlements can only be permanently uplifted by repairing and regenerating the environment in which people live. It is an integrated socio-technological intervention that centres around communities as active participants in the design and implementation of a customised suite of elements tailored to the circumstances and needs of their respective settlements. These elements, designed at the communal neighbourhood scale, include: (i) the provision of new toilets or renovations for households in need and their connection to onsite septic treatment; (ii) decentralized, nature-based treatment systems (wetlands) for wastewater or the direct connection of septic waste systems to mains sewage in order to minimise sewage pollution within the settlement and receiving waterways; (iii) drainage controls to mitigate flooding, ponding and external contamination; (iv) pathways for resilient all-weather access to dwellings and flood evacuation; and (v) other structural resilience and pollution abatement controls as needed for each location. Together, these elements are designed to work with the available space contraints of the settlement area, and protect and enhance environmental values. The intervention is an 'acupunctural', site-specific, nature-based upgrade designed to be implementable without changes to residence and without relocation or other impositions.

Led by Monash University, RISE was initiated in 2017 as a 5-year program with research funding from the Wellcome Trust. The neighbourhood upgrades in Suva are funded by the Fiji and New Zealand governments, and the upgrades in Indonesia by the Australian government. Due to Covid and other logistic delays, the program was extended by 4 years to conclude in 2026.

Construction of neighbourhood upgrades in the intervention settlements was completed in Makassar in 2024, and due for completion in Fiji in early 2026. The RCT has involved regular campaign-based surveys of resident health and wellbeing (self-reported), child anthropometry, child stool and blood collection, soil and water sampling, and ecological and environmental monitoring. The program

involves comprehensive genomic and metagenomic analyses of human and environmental samples for detection of *E. coli* and other pathogens as well as for antimicrobial resistance genes, focusing on evaluating any decrease in environmental enteric pathogen contamination as a consequence of the interventions.
